# Thromboelastometry-guided hemostatic therapy for hemorrhagic shock in the postoperative period of vascular surgery: a case report

**DOI:** 10.1186/s13256-018-1661-8

**Published:** 2018-06-02

**Authors:** Tomaz Crochemore, Felicio A. Savioli

**Affiliations:** Hospital Israelita Albert Einstein – Intensive Care Unit, Av. Albert Einstein, 627, Morumbi, São Paulo, SP CEP: 05651-901 Brazil

**Keywords:** Thromboelastography, Thromboelastometry, Viscoelastic tests, Hemorrhagic shock, Vascular surgery, Postoperative bleeding, Hemostatic therapy

## Abstract

**Background:**

Hemorrhagic shock is a medical emergency that often complicates vascular surgery and can lead to death. Hemorrhagic shock is characterized by hypoperfusion and hemodynamic abnormalities leading to the collapse of homeostasis due to massive blood loss. Early diagnosis is critical for a favorable outcome. Thromboelastometry has been considered an effective tool for bleeding management in critically ill patients. Thromboelastometry can guide transfusion therapy quickly, reducing the need for blood products. Therefore, it could be an alternative test to guide hemostatic therapy in complex cases of hemorrhagic shock as a result of vascular surgeries. We report our successful experience with a case of hemorrhagic shock in postoperative care in vascular surgery, in which bleeding management was guided by thromboelastometry and bleeding control was achieved with hemostatic drugs and coagulation factor concentrates.

**Case presentation:**

We report a case of an 82-year-old Afro-Brazilian man who presented to the intensive care unit with hemorrhagic shock in the postoperative period of vascular surgery. He underwent surgery for correction of iliac artery aneurysm with endoleak. His laboratory tests revealed severe anemia (hemoglobin 7.4 mg/dL), metabolic acidosis (bicarbonate 10 mEq/L, pH 7.11), acute kidney injury (creatinine 3.1 mg/dL), thrombocytopenia (platelets count 83 × 10^3^/mm^3^), hypofibrinogenemia (70 mg/dL), international nationalized ratio 1.95, activated partial thromboplastin time 64.5 seconds, and lactate 87 mmol/L. There was active bleeding in surgical site. Bleeding management was guided by thromboelastometry. The first test showed fulminant hyperfibrinolysis, which was corrected with the administration of tranexamic acid. The second thromboelastometry test showed improvement of hyperfibrinolysis but severe hypocoagulability. Fibrinogen concentrate, platelet apheresis, cryoprecipitate, and prothrombin complex concentrate were sequentially administrated. Thromboelastometry was completely corrected after 2 hours. Arteriography to evaluate mechanical cause of bleeding was normal. No more bleeding was identified, and neither was any further transfusion needed. He was discharged from the intensive care unit from the ward 3 days after admission.

**Conclusions:**

Thromboelastometry may be considered a useful, feasible and safe tool to monitor and manage coagulopathy in patients with hemorrhagic shock. Moreover, it has the potential benefit of allowing rapid diagnosis,  goal-directed therapy with hemostatic drugs and coagulation factor concentrates and thus, avoiding unnecessary blood component transfusion.

## Background

Hemorrhagic shock is a medical emergency related to surgery, invasive procedures, and trauma. Ongoing severe bleeding is associated with increased morbidity and mortality, and may promptly require additional surgery. Early diagnosis of hemorrhagic shock is critical for a favorable outcome [1, 2]. Hemorrhagic shock represents a clinical condition of impaired tissue perfusion resulting from acute reduction in intravascular blood volume, hemodynamic instability, decreased oxygen delivery, cellular hypoxia, organ dysfunction, and death [3]. Resuscitation involves interrupting the source of bleeding and restoring the circulation of blood volume. Fluids are the first-line therapeutic option to restore intravascular blood volume but there is still a lack of consensus in the literature concerning the optimal fluid used in patients presenting hemorrhagic shock. Fluids should be administered to improve perfusion parameters such as blood pressure, urine output, or lactate [4]. Anemia should be promptly corrected with red blood cells (RBC) to restore blood loss [5]. However, quantifying blood loss can be a difficult task. Transfusion of allogeneic blood components is necessary when the estimated blood loss from hemorrhage exceeds 30% of blood volume (class III hemorrhage) [1]. A hemoglobin level of 7–8 g/dl has been considered an appropriate threshold for transfusion in critically ill patients with no risk factors for tissue hypoxia [5]. Maintaining hemoglobin level between 9 and 10 g/dl seems a reasonable goal for patients with active bleeding or with high risk of myocardial infarction. The ability to quickly diagnose hemorrhagic shock is critical and necessary for favorable outcomes. The clinical setting is usually characterized by: severe hypotension (systolic blood pressure < 90 mmHg); tachycardia, that is, heart rate > 120 beats per minute (bpm); metabolic acidosis (blood lactate > 2–3 mmol/L or base deficit < 4 mmol/L); decreased pulse pressure; cold skin; and impaired consciousness [6].

Traditional therapeutic options for bleeding control associated with hemorrhagic shock include transfusion of RBC and allogeneic blood products, such as fresh frozen plasma (FFP), platelet concentrates (PC), and cryoprecipitate (Cryo). Nowadays, coagulation factor concentrates and hemostatic drugs have been considered a good alternative since blood components transfusion is associated with serious adverse events and increased mortality [7]. Hemostatic therapy might be empiric in fixed ratio, based on conventional coagulation tests (CCT) or guided by point-of-care testing (POCT). The main goal in the management of hemorrhagic shock should be bleeding control without increasing the risk of thrombotic events.

There is a lack in scientific literature of high quality evidence concerning efficacy of the traditional practice of transfusion in fixed ratio of RBC and FFP of 1:1 to treat life-threatening bleeding. In addition, traditional transfusion with blood components does not focus on the specific disorders of coagulation [8, 9]. Furthermore, currently there is a trend in major centers in Europe to avoid the use of blood components due to the high risk of adverse events. Possible complications associated with transfusion of blood components include transfusion-related acute lung injury (TRALI), transfusion associated circulatory overload (TACO), nosocomial infections, sepsis, transfusion-related immunomodulation (TRIM), and organ dysfunction [10, 11].

CCT such as prothrombin time (PT)/international nationalized ratio (INR) and activated partial thromboplastin time (aPTT) are poor predictors of bleeding. CCT fail to identify specific coagulation disorders such as hyperfibrinolysis, hypercoagulability, and the platelet component [12]. In fact, CCT were originally indicated to monitor anticoagulant drugs including heparin and warfarin. On the other hand, POCT such as thromboelastometry (ROTEM™) has been associated with a reduction in the need for blood component transfusion in different clinical settings [13]. Thromboelastometry allows rapid identification of a specific coagulation disorder in order to predict early massive transfusion and guide goal-directed therapy with specific hemostatic drugs and coagulation factor concentrates [14, 15]. These results were recently confirmed by a prospective randomized clinical trial in cardiac surgery demonstrating a significant reduction in transfusion requirements, transfusion-associated adverse events, costs, as well as improved outcomes including 6-month mortality in the point-of-care group compared to the control group [16].

We aimed to report an interesting case where bleeding management associated with hemorrhagic shock in the postoperative period of vascular surgery was successfully reversed guided by ROTEM at the bedside. Thromboelastometry enabled the early identification of specific coagulation disorders associated with hemorrhagic shock and, therefore, guided hemostatic therapy by goals.

## Case presentation

We report the case of an 82-year-old Afro-Brazilian man (weight 72 kg) who presented to the intensive care unit (ICU) in the immediate postoperative period of elective surgery with signs of hemorrhagic shock. This surgery was performed to treat iliac artery aneurysm with endoleak type 1 to 1B with placement of Endurant™ IIS trimodular prosthesis. His previous medical history included dementia, arterial hypertension, tobacco smoking, ischemic heart disease, and aortic insufficiency. He denied consuming alcohol. At home, he was stable and felt well. He was calm and performed his daily activities under medical supervision, despite early stage dementia. He had no motor deficit, only idle speed typical of the elderly. His blood pressure was controlled, with no clinical signs of heart failure. According to his family, he had a good quality of life. Three months earlier he had undergone vascular surgery to treat an aneurysm in his common iliac artery with endoprosthesis. The surgery had no complications and he was rehabilitated at home. However, 3 days prior to hospital admission he had a computed tomography scan. The result of imaging showed an increase in aneurysm size in his common iliac artery. Then a new surgery was indicated for the treatment of the aneurysm.

The present surgery was performed under general anesthesia, with hemodynamic instability throughout the procedure, which required norepinephrine. Iliac dissection technique was difficult; there was loss of blood and a need for suture and compressive dressing. Cefuroxime was administered intravenously in the operating room at 1.5 g intravenously and maintained every 8 hours postoperatively. In addition, 3500 ml of crystalloid, and 1 unit of RBC were administered. Our patient left the surgery without a distal pulse; 31 mL of contrast was needed. He presented hypotension and alteration of consciousness after anesthesia wore off. His low blood pressure was increased with metaraminol use, a sympathomimetic drug that produces peripheral vasoconstriction (compresses peripheral vessels) by direct action on alpha-adrenergic receptors. An infusion of norepinephrine was needed after initial recovery of blood pressure. After his blood pressure had improved and he regained consciousness, the anesthesiologist opted for extubation in the operation room. At the ICU, he presented fast clinical worsening. He showed an altered level of consciousness (Glasgow Coma Scale of 9), agitation, delirium, and mental confusion. His arterial blood pressure was 70/35 mmHg, heart rate was 145 bpm, and respiratory rate 42 breaths per minute. He presented active bleeding in the femoral region, at the arterial puncture site. Hypothermia (34 °C), sweating, oliguria, bleached mucosa, cold extremities, and signs of severe tissue hypoperfusion completed the clinical scenario. The clinical picture was strongly suggestive of hemorrhagic shock. Laboratory tests revealed acidosis and severe anemia with hemoglobin of 7.4 g/dL. CCT were greatly altered, platelets count 83 × 10^3^/mm^3^, plasma fibrinogen concentration 70 mg/dL, and prolonged INR 1.95. General blood tests showed total bilirubin 1.1 mg/dL, serum creatinine 3.1 mg/dL, serum aspartate aminotransferase (AST) 48 U/L, serum alanine aminotransferase (ALT) 32 U/L, arterial bicarbonate 10 mEq/L, arterial pH 7.11, blood glucose 98 mg/dL, ionic calcium 1.02 mmol/L, and lactate 87 mg/dl. Central vein catheterization and endotracheal intubation were performed due to circulatory shock, consciousness impairment, and airway patency protection. Our patient received an initial fluid load of 3000 mL of 0.9% saline solution. He was warmed and calcium gluconate was administered. An initial transfusion therapy with 4 units of RBC was urgently performed according to estimated blood loss. Norepinephrine infusion was associated due to hemodynamic instability to maintain a mean blood pressure above 65 mmHg. The coagulopathy treatment was performed guided by ROTEM.

**Fig. 1:**
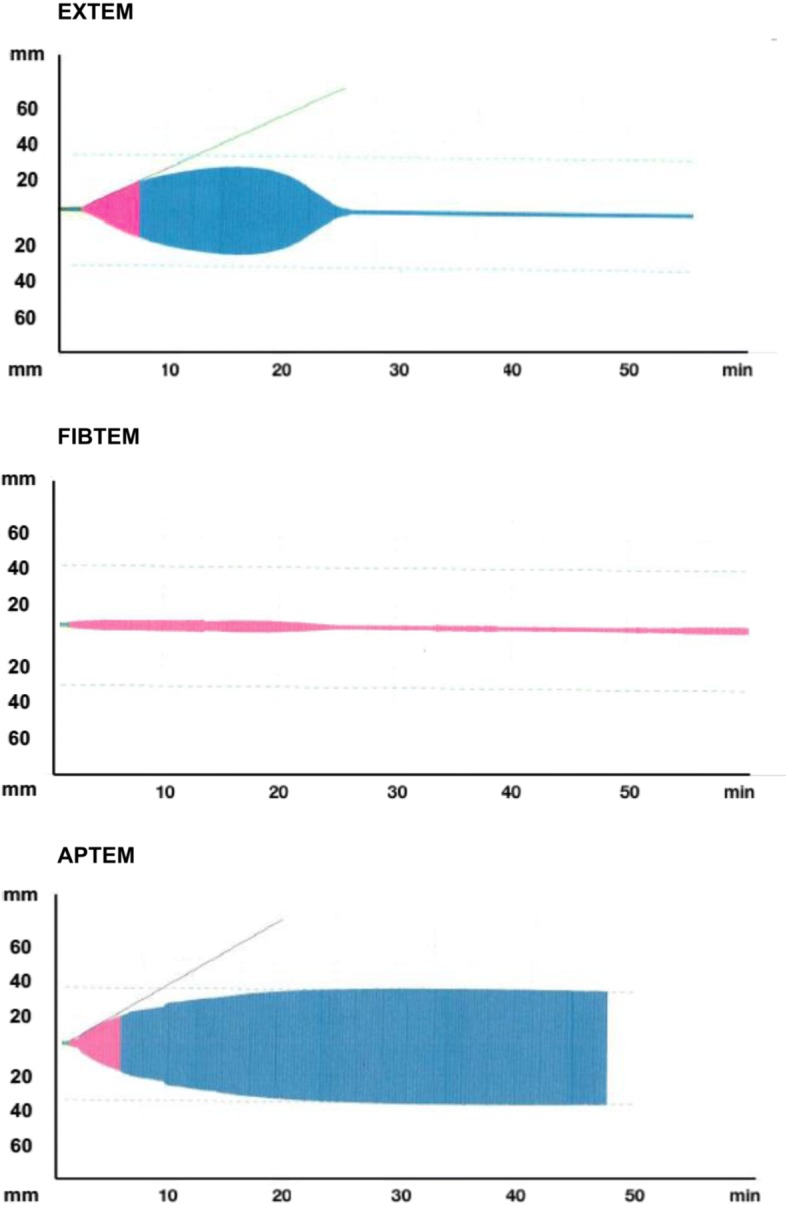
First Thromboelastometry (ROTEM®) analysis in intensive care unit. Both EXTEM (extrinsic coagulation pathway) and FIBTEM (cytochalasin D) tests showing fulminant hyperfibrinolysis. APTEM (aprotinin) is normal. APTEM uses aprotinin to inhibit fibrinolysis

**Fig. 2:**
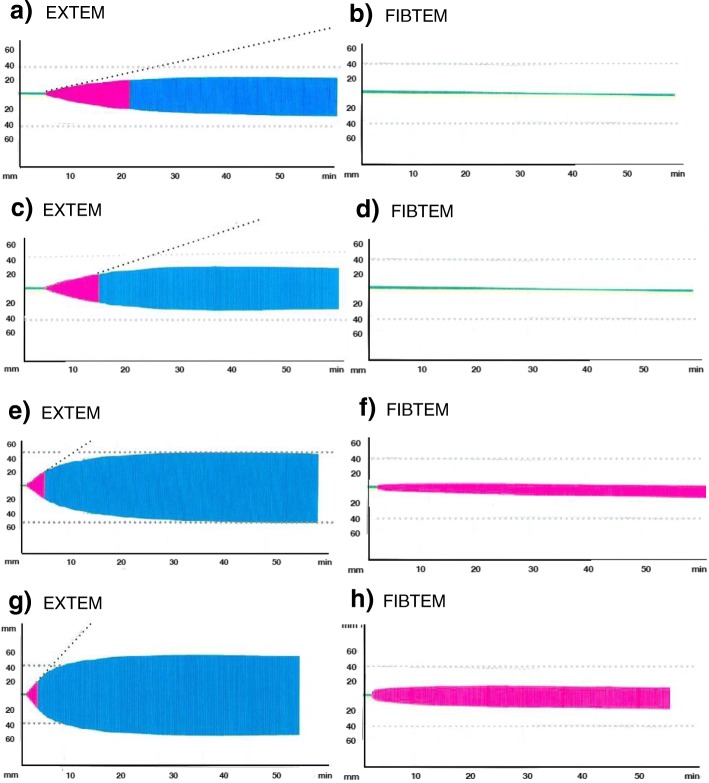
**a**-**b**) Second Thromboelastometry analysis (ROTEM®) 30 minutes after tranexamic acid showing kinect and structural hypocoagulability in both EXTEM (extrinsic coagulation pathway) and FIBTEM (cytochalasin D); **c**-**d**) Third Thromboelastometry analysis showing prolonged clotting time in EXTEM and maximum clot firmness reduced in FIBTEM; **e**-**f**) Fourth Thromboelastometry analysis corrected and bleeding was controlled; **g**-**h**) Thromboelastometry analysis control

A ROTEM (ROTEM®; Pentapharm Co., Munich, Germany) was performed in the immediate postoperative period in the ICU. The first thromboelastometry analysis showed fulminant hyperfibrinolysis in the presence of active bleeding. The FIBTEM revealed severe impairment in the fibrinogen function (Fig. 1). Based on maximum lysis (ML = 99%) in EXTEM, 1.0 g of tranexamic acid was administered in bolus. A second thromboelastometry analysis was performed 30 minutes after tranexamic acid infusion due to continuous bleeding, showing a severe hypocoagulation in EXTEM with maximum clot firmness (MCF) EXTEM 32 mm and FIBTEM with MCF FIBTEM 4 mm (Fig. 2a, b). Based on these tests, 6.0 g of fibrinogen concentrate (FC; Haemocomplettan® P; CSL Behring, Marburg, Germany) and 1 platelet apheresis were administered. A third ROTEM was performed due to persistent bleeding, showing a prolonged clotting time (CT) in EXTEM and reduced MCF in FIBTEM (Fig. 2c, d). According to the third ROTEM, 9 units of Cryo and 1500 UI of prothrombin complex concentrate (PCC; Beriplex ® P/N 500 UI; CSL Behring, Marburg, Germany) were administered. The last thromboelastometry was completely corrected after 2 hours of treatment, and bleeding was controlled (Table 1 and Fig. 2e, f). Our patient presented clinical and hemodynamic stability with dose reduction of norepinephrine. Arteriography performed to evaluate mechanical cause of bleeding was normal.

**Table 1:** Evolution of thromboelastometry parameters

Time points	Assays	CT (seconds)	CFT (seconds)	A10 (mm)	MCF (mm)	ML (%)
0 minute	EXTEM	111	323	28	32	99
	FIBTEM	96		4	4	66
	APTEM	134	311	29	41	2
30 minutes	EXTEM	302	957	16	26	0
	FIBTEM	4085			0	0
60 minutes	EXTEM	252	601	20	31	9
	FIBTEM	3674			0	0
90 minutes	EXTEM	78	219	36	50	5
	FIBTEM	86		6	7	0
120 minutes	EXTEM	80	145	44	56	3
	FIBTEM	77		11	14	2

*A10* clot formation after 10 minutes, *CT* clotting time, *CFT* clot formation time, *MCF* maximum clot firmness, *ML* maximum lysis, *EXTEM* extrinsic coagulation pathway, *INTEM* intrinsic coagulation pathway, *FIBTEM* cytochalasin D, *HEPTEM* Heparinase, *APTEM* aprotinin

No active bleeding was identified. Hemodialysis was started within 24 to 36 hours. There was no additional need for blood components transfusion on subsequent days. He was discharged from our ICU and cleared for a ward 3 days after admission. No new organ dysfunction or infection was identified, and the result of the microbiology was negative. He was transferred to a ward for clinical rehabilitation. At follow-up, he required conventional hemodialysis for 5 days. He was treated for pneumonia with piperacillin-tazobactam, oxygen supplementation, and respiratory and motor physiotherapy for 7 days. With improved renal function and recovery of urinary output, and with no other signs of infection, he was discharged after 2 weeks in hospital. At follow-up after 6 months, he was at home, in rehabilitation with physiotherapy, without dialysis or infection.

## Discussion

We report this case of hemorrhagic shock complicating postoperative vascular surgery for treatment of common iliac artery aneurysm. Our patient presented clinical signs of low cardiac output and tissue hypoperfusion, including hypotension, mental alteration, and cold extremities, in the presence of active bleeding at a surgical site in the right femoral region. Advanced life support with orotracheal intubation for airway protection and partial hemodynamic stabilization with crystalloids and RBC were immediately performed. He was warmed and given calcium gluconate and calcium bicarbonate for biochemical balance. Bleeding management was successfully guided by ROTEM at the bedside. Different clotting disorders such as hyperfibrinolysis, hypofibrinogenemia, platelet disease, and clotting factor deficiency were identified, sequentially. As a result, thromboelastometry allowed specific treatment with hemostatic drugs, coagulation factor concentrates, and even allogeneic blood components.

Hemorrhagic shock is an important cause of mortality in surgical patients; it is responsible for more than 80% of deaths in the operating room [1]. Hemorrhagic shock is a medical emergency characterized by inadequate tissue perfusion resulting from an acute decrease in intravascular blood volume leading to hypovolemic state. The clinical picture includes severe hypotension (systolic blood pressure < 90 mmHg), tachycardia (heart rate > 120 bpm), metabolic acidosis (blood lactate > 2–3 mmol/L or base deficit < 4 mmol/L), decreased pulse pressure, cold skin, sweating, and altered state of consciousness, including drowsiness, torpor, disorientation, or confusion [8].

Multiple factors are considered a cause of bleeding in surgical patients, including blood loss, hemodilution, fibrinogen dysfunction, acquired platelet dysfunction, coagulation factor consumption, activation of fibrinolytic system, and hypothermia. Antiplatelet and anticoagulant drugs can lead to coagulopathy during surgery complicating bleeding control. Surgical or mechanical bleeding may occur due to an uncontrolled source in the absence of coagulopathy [9]. Immediately after tissue damage during major surgery, the exposure of the thromboplastin-rich subendothelial tissue to flowing blood induces the activation of coagulation and an initial hypercoagulable state [10]. Uncontrolled bleeding leads to loss of coagulation factors and a later decrease in platelet counts. Hypovolemia due to intravascular blood volume loss and shock leads to tissue hypoperfusion and endothelial dysfunction [11]. Thrombomodulin (TM) expressed on vascular endothelium binds to thrombin forming a complex and it acts as an anticoagulant. In addition, the thrombin-TM complex activates protein C to produce activated protein C (APC), which inactivates factors VIIIa and Va in the presence of protein S, thereby inhibiting further thrombin formation: a hypocoagulable state [12]. Tissue injury in surgery may lead to the exposure of tissue plasminogen activator by the endothelium, resulting in hyperfibrinolysis and impairing hemostasis, thereby contributing to the exacerbation of bleeding [4]. Dilution of coagulation factors and platelets by fluids resuscitation and blood transfusion aggravates coagulopathy [14]. Hypothermia, which slows down enzymatic reactions, modifies platelet function, decreases platelet counts, and stimulates fibrinolysis. Acidosis worsens fibrin polymerization and impairs clot strength. Low-ionized calcium due to massive RBC transfusions containing citrate and low hematocrit further aggravates bleeding diathesis [15, 17].

The priority treatment of hemorrhagic shock is to control the source of bleeding as quickly as possible and to replace fluid and improve hemodynamics parameters maintaining oxygen delivery to limit tissue hypoxia, inflammation, and organ dysfunction. There is a lack of literature on what kind of fluid is superior to other fluids. Although colloids could induce a more rapid and persistent plasma expansion because of a larger increase in oncotic pressure, colloids may impair the coagulation system, decreasing factor VIII activity, von Willebrand factor (vWF) function, and fibrin polymerization [18]. On the other hand, resuscitation with large volumes of crystalloids has been associated with tissue edema, an increased incidence of abdominal compartment syndrome, and hyperchloremic metabolic acidosis [19, 20]. The resuscitative strategy still involves fluid resuscitation and the use of vasopressors and blood transfusion to prevent or correct coagulopathy [21].

Currently, there is growing evidence in the literature showing that exposure to allogeneic blood transfusion has been associated with serious adverse events, such as TRALI, TACO, nosocomial infections, sepsis, TRIM, and organ dysfunction [22]. FFP was associated with an increased risk of infection in surgical patients [23]. Blood transfusion can lead to pulmonary complications, which are responsible for the majority of morbidity and mortality, associated with blood component transfusion in hospitalized patients [24].

CCT were originally intended to monitor the effect of anticoagulant drugs. Despite their extended use to identify coagulopathy, CCT are weak predictors of bleeding in critically ill patients. *In vivo* coagulation system occurs primarily on the surface of platelets. Tissue factor-bearing cells and RBC also play a significant role in hemostasis. In the absence of blood cells, which are removed by centrifugation, CCT are performed using plasma. Also, these tests are stopped upon formation of the first fibrin strands when only 5% of the total thrombin has been generated [8, 25]. Moreover, conventional coagulation tests do not assess the quality and the strength of clot and fail to identify hypercoagulability, hyperfibrinolysis, and platelet component.

In contrast, viscoelastic tests are performed in whole blood sample providing a better reflection of the *in vivo* coagulation, considering interaction between blood cells, soluble coagulation factors, and platelets. Viscoelastic tests, such as ROTEM or thromboelastography (TEG) have been associated with a reduction in the need for blood components transfusion in critically ill patients in different clinical situations, including cardiac surgery, trauma, liver transplantation, and postpartum hemorrhage [26]. Viscoelastic tests allow global dynamic information about clot formation process with rapid results. ROTEM can predict massive transfusion, identifying the cause of coagulopathy. As a result, thromboelastometry can guide goal-directed therapy with specific hemostatic drugs, coagulation factor concentrates, and allogeneic blood products. Reagents tests such as EXTEM (extrinsic coagulation pathway activated by thromboplastin or tissue factor) and INTEM (intrinsic coagulation pathway activated by contact phase – ellagic acid) are used for initial screening to identify the presence of coagulopathy. FIBTEM (cytochalasin D), HEPTEM (heparinase), and APTEM (aprotinin) tests are used to identify a specific diagnosis of coagulopathy, and thus guide transfusion therapy according to the needs of each patient. The parameters analyzed for the interpretation of the thromboelastometry curve, as in the EXTEM, include CT (in seconds), coagulation formation time (CFT) in seconds, MCF (in mm), and ML (in percentage). CT demonstrates the period related to the initial process of clot formation and fibrin polymerization. A prolongation in CT suggests deficiency of coagulation factors or heparin effect. CFT represents the period from thrombin generation to MCF, which is determined mainly by fibrinogen, platelets, and factor XIII. A prolongation in CFT indicates a hypofibrinogenemia, platelet dysfunction, or factor XIII deficiency. MCF demonstrates clot strength. Changes in MCF can identify a state of hypercoagulability or hypocoagulability. Finally, ML demonstrates the last period of clot stabilization. ML reduced suggests the presence of hyperfibrinolysis, which can be confirmed comparing EXTEM with APTEM. ML over 15% in EXTEM that is corrected by aprotinin (APTEM test) confirms the diagnosis of hyperfibrinolysis [27].

This case report describes a patient with hemorrhagic shock in the immediate postoperative period of vascular surgery with signs of active bleeding at the surgical site in inguinal region. The initial approach included transfusion of 4 units of RBC and fluid replacement with crystalloid and norepinephrine to achieve mean blood pressure around 65 mmHg and preserve tissue perfusion. Our patient underwent orotracheal intubation and a central venous access in deep jugular vein was inserted. Acidosis and hypocalcemia were corrected and he was heated. Transfusion therapy was guided by ROTEM. An initial ROTEM examination showed early fulminant hyperfibrinolysis (ML in EXTEM greater than 15% and ML normal in APTEM test). Unlike CCT, ROTEM allowed an early diagnosis of this coagulopathy, within the first 5 to 10 minutes. Administration of 1 g of tranexamic acid, a low-cost drug in bolus infusion, quickly corrected the severe coagulation disorder. Thirty minutes after administration of the antifibrinolytic drug, the second ROTEM showed a correction of the hyperfibrinolysis, but a severe hypocoagulability (decreased MCF in EXTEM) in the presence of active bleeding was identified. In that moment, 6 g of FC and, after that, 1 platelet apheresis were administered. Thirty minutes later, the sequential ROTEM test still showed a serious state of hypocoagulability, a delayed thrombin generation process, and maintenance of hemorrhage. At this time, 9 units of Cryo were administered to correct clot strength and 1500 units of PCC to correct the initial phase of thrombin generation. Finally, there was clinical bleeding control, 30 minutes after using Cryo and PCC, guided by ROTEM. In this case, ROTEM became normal 120 minutes after the first ROTEM with hyperfibrinolysis.

Some aspects of this transfusion practice for the correction of coagulopathy should be highlighted. The order or sequence of the therapeutic approach in this scenario of bleeding management is very important and can make a difference for the patient. First of all, ROTEM is a POC testing performed at the bedside. Instead of coagulation tests, ROTEM allows rapid assessment of the whole clot formation process with early identification of disturbance of coagulopathy. In this reported case, ROTEM identified hyperfibrinolysis, and thus guided the specific therapy with hemostatic drug (tranexamic acid). In severe acute bleeding management, the first step is to correct the clot stabilization process by administering an antifibrinolytic drug. Otherwise, continued consumption of fibrinogen should be maintained. The second step involves the correction of MCF or clot strength, determined by fibrinogen, platelets, and factor XIII. In this case, even after 6 g of FC, there was persistence in the hypocoagulability, which was corrected after transfusion of PC, but mainly with transfusion of Cryo, composed of fibrinogen but also factor XIII. The last step includes improving the initial thrombin generation process, through replacement of coagulation factors or reversal of the effect of present heparin.

In hemorrhagic shock, coagulation disorder is complex, dynamic, and variable over time. CCT take 45 to 60 minutes to read. CCT are unable to identify coagulation disorders contemporaneously as in this reported case, that is, the presence of hyperfibrinolysis. The rapid interpretation of ROTEM associated with the use of hemostatic drugs and coagulation factor concentrates allows a more precise, individualized, and early treatment of bleeding. Traditional transfusion practice with blood components such as RBC, FFP, Cryoprecipitate, and PC, requires time for blood typing, thawing, and transportation from the blood bank. This delay in administration of blood component may be a determining factor for an unfavorable outcome. Similarly, the empirical use of blood components in the absence of ROTEM increases the risk of adverse effects to the patient and may fail to correct the mechanism responsible for bleeding.

## Conclusions

In summary, successful bleeding management of patients with hemorrhagic shock is still a challenge for anesthesiologists. Early recognition of the specific cause of coagulopathy followed by appropriate treatment is associated with a favorable outcome. Thromboelastometry may be considered a useful, feasible, and safe tool for rapid diagnosis of different coagulation disorders associated with hemorrhagic shock. Thromboelastometry still presents the potential benefit of allowing goal-guided hemostatic therapy with hemostatic drugs and coagulation factor concentrates. Thus, this POCT could help avoid unnecessary transfusion in patients with hemorrhagic shock and active bleeding. Additional studies are expected to define the role and benefit of the use of thromboelastometry for diagnosis of coagulopathy and as a guide for transfusion therapy with allogeneic blood products, coagulation factor concentrates, or hemostatic drugs.

## Abbreviations

ALT: Serum alanine aminotransferase
APC: Activated protein C
aPTT: Activated partial thromboplastin time
AST: Serum aspartate aminotransferase
bpm: beats per minute
CCT: Conventional coagulation tests
CFT: Clot formation time
Cryo: Cryoprecipitate
CT: Clotting time
FC: Fibrinogen concentrate
FFP: Fresh frozen plasma
ICU: Intensive care unit
INR: International nationalized ratio
MCF: Maximum clot firmness
ML: Maximum lysis
PC: Platelet concentrates
PCC: Prothrombin complex concentrate
POCT: Point-of-care testing
PT: Prothrombin time
RBC: Red blood cells
ROTEM: Thromboelastometry
TACO: Transfusion associated circulatory overload
TEG: Thromboelastography
TM: Thrombomodulin
TRALI: Transfusion-related acute lung injury
TRIM: Transfusion-related immunomodulation
vWF: Von Willebrand factor
